# New Evaluation Method for Bone Formation around a Fully Hydroxyapatite-Coated Stem Using Digital Tomosynthesis: A Retrospective Cross-Sectional Study

**DOI:** 10.3390/diagnostics11112094

**Published:** 2021-11-12

**Authors:** Sho Totsuka, Tomofumi Nishino, Ryunosuke Watanabe, Masashi Yamazaki, Hajime Mishima

**Affiliations:** Department of Orthopaedic Surgery, Faculty of Medicine, University of Tsukuba, Tsukuba 305-8575, Japan; s.totsuka0705@tsukuba-seikei.jp (S.T.); nishino@md.tsukuba.ac.jp (T.N.); ryuwatanabe@tsukuba-seikei.jp (R.W.); masashiy@md.tsukuba.ac.jp (M.Y.)

**Keywords:** POLARSTEM, fully hydroxyapatite-coated stem, digital tomosynthesis, surrounding bone formation

## Abstract

Digital tomosynthesis (DTS) is a new imaging technique derived from radiography, and its usefulness has been gradually reported in the field of orthopedic diagnosis in recent years. A fully hydroxyapatite (HA)-coated stem, which is used for total hip arthroplasty (THA), is a type of cementless stem that has been widely used recently and reported to have good results. However, stem loosening on plain radiographs is difficult to determine in some cases due to cancellous condensation around the stem. In this retrospective cross-sectional study, we compared the results of plain radiography versus DTS to evaluate the imaging findings after THA using a fully HA-coated stem. Twenty joints each in the 3 y and 1 y postoperative groups underwent plain radiography and DTS. On DTS, bone formation around the stem was confirmed in all cases; however, this formation was not reproducible on plain radiography, and there were cases in which the reaction could not be confirmed or cases with cancellous condensation resembling reactive lines. This reaction was not reproducible on plain radiographs, and in some cases, the reaction could not be confirmed, or there were cases with cancellous condensation that resembled reactive lines. Therefore, DTS was useful in the diagnosis of bone formation around the implant.

## 1. Introduction

Digital tomosynthesis (DTS) is a new imaging technology derived from radiography, and various clinical applications of DTS have been reported [1,2,3]. Developed in the 1980s, it was initially used as an adjunct to mammography. However, after the development of a digital fluoroscopy table equipped with a flat panel detector in 2004, it has since become widely used, and in recent years, research on its application in musculoskeletal imaging has been conducted. While conventional tomography requires a large amount of radiation to obtain a single cross-section, DTS can generate a series of images from images projected from various angles in a single shot, thereby requiring a much lower radiation dose than conventional tomography [3,4].

Fully hydroxyapatite (HA)-coated stems are a type of cementless stem that have been widely used recently and are reported to have good long-term results [5]. HA-coated stems exhibit radiographic silence with less evidence of bone reactions, such as reactive lines (RLs) and cortical hypertrophy [6,7]. However, among 3 y postoperative cases of total hip arthroplasty (THA) using POLARSTEM (Smith & Nephew, Inc., Memphis, TN, USA), there were some cases with cancellous condensation, which were difficult to distinguish from RLs upon first glance on plain radiography.

**Figure 1:**
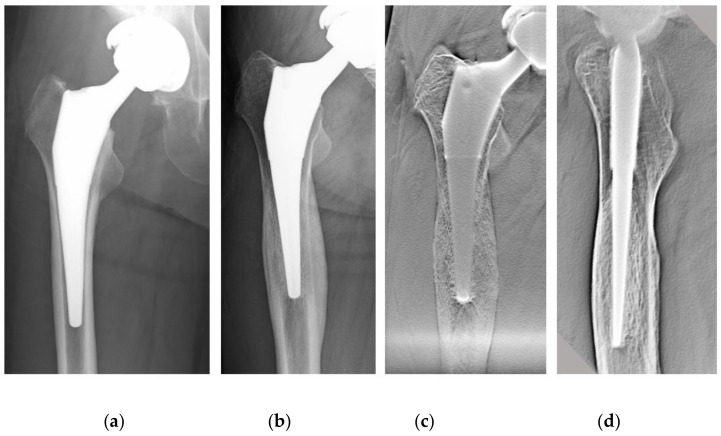
A 61-year-old female patient after THA using a tapered wedge stem. (**a**) Plain radiographs one month after THA. (**b**) The same patient 6 y later. There was cortical hypertrophy at Gruen Zones 3 and 5. (**c**) DTS (AP view) at the same time as (**b**). Cortical hypertrophy was observed more clearly. (**d**) DTS (lateral view) at the same time as (**b**). Heterogeneous loss of permeability is seen in the cancellous bone distal to the porous coating. THA, total hip arthroplasty; DTS, digital tomosynthesis; AP, anterior–posterior.

The loosening of the prosthesis may not be visible either clinically or by imaging, and reliable imaging methods are needed for early diagnosis [8,9]. Plain radiography and computed tomography (CT) are the most widely used methods for evaluating bone reactions around cementless stems [10,11]. However, plain radiography is difficult to reproduce owing to the inconsistent body position and the lack of spatial resolution in the depth direction, making detailed image evaluation difficult [12,13]. CT also distorts the bone–implant interface due to metal artifacts, and the radiolucent line (RLL) may be underestimated or not identified [14]. Therefore, we focused on DTS to evaluate the stem in detail. We used DTS to diagnose hip loosening and evaluate cortical hypertrophy and biological fixation and applied it to preoperative planning for THA revision (Figure 1).

**Figure 2:**
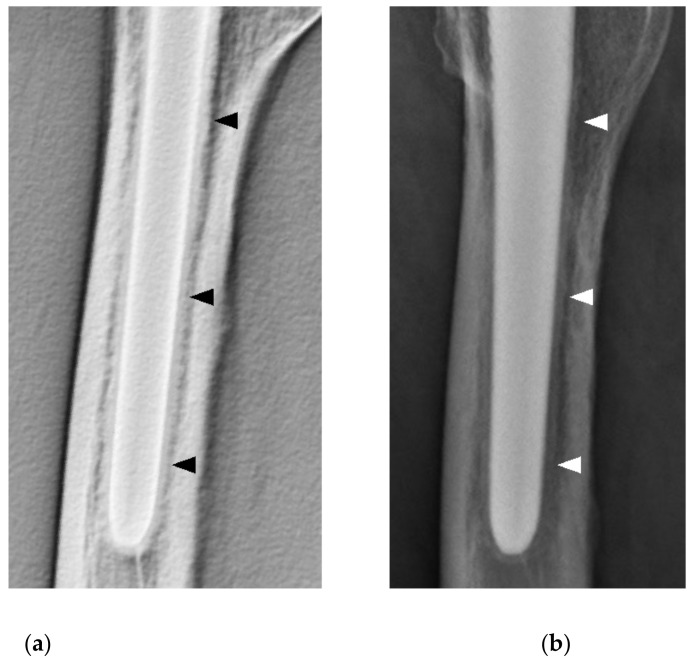
An 83-year-old female patient 3 y after THA. (**a**) DTS (lateral view); there was bone formation surrounding the stem (black arrowheads). (**b**) Plain radiographs (lateral view) of the same patient as in (**a**); there was cancellous condensation similar to a reactive line (white arrowheads). THA, total hip arthroplasty; DTS, digital tomosynthesis.

This time, DTS of a case with cancellous condensation revealed bone formation around the stem (Figure 2).

The evaluation of RLs and spot welds using DTS in the hip region has been shown to be useful [15,16,17,18]. However, studies on bone response specific to various stem geometries are scarce, and there are no reports on the evaluation of a fully HA-coated stem using DTS. Therefore, this study aimed to perform detailed observations around the fully HA-coated stem using DTS, evaluate the usefulness of DTS in THA, determine whether there is a specific response, and examine short-term changes.

## 2. Materials and Methods

### 2.1. Study Design and Patients

**Figure 3:**
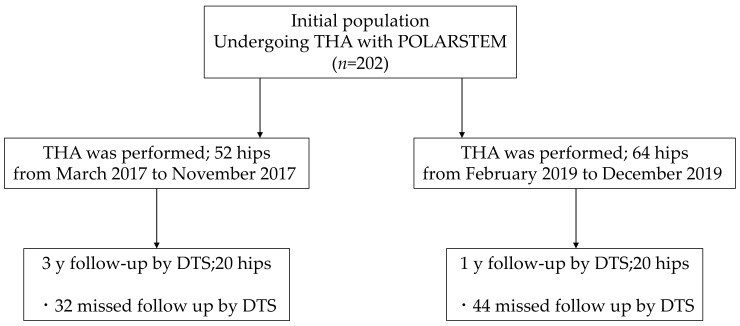
Flow diagram of the study design.

Patients who underwent primary THA with the POLARSTEM, described later, at our institution were included in this cross-sectional study. Patients who had undergone previous surgery on the same joint were excluded (Figure 3).

Morphological evaluation of the hip was conducted using preoperative images from plain anteroposterior radiographs of the pelvis and both hips. According to the Dorr classification, the femurs were classified as either Type A (champagne flute shape), B (funnel shape), or C (cylindrical shape). The Canal Flare Index (CFI) is defined as the ratio of the diameter of the femoral canal at the isthmus in the anteroposterior view to the diameter of the medullary canal 2 cm above the lesser trochanter [19].

### 2.2. Digital Tomosynthesis

We used the EXAVISTA system (Hitachi, Ltd., Tokyo, Japan). DTS is a function that creates coronal tomographic images by using a cone-beam CT reconstruction algorithm based on the projection data obtained using continuous imaging from multiple directions while changing the angle of the fluoroscopy table imaging system. Only the cross-sections of interest can be displayed from multiple reconstructed tomographic images to avoid overlap in the depth direction for diagnosis. The EXAVISTA system can display tomographic images in approximately 30 s, including the acquisition time. Additionally, because projection images are acquired and reconstructed at a size of 1 × 1 px, high-resolution images can be obtained. DTS not only reconstructs the projection image, but also provides a unique image with the following processing.

#### 2.2.1. Thickness Addition Processing

The thickness addition process adds the weights of the previous and next images to the reconstructed tomographic image, thereby displaying an image with reduced noise while maintaining the sharpness of the edge of the X-ray hyperabsorbing contour. This weighting was optimized according to the region to obtain the best image for each examination [20].

#### 2.2.2. Metal Artifact Reduction Technology (High-Quality Metal Artifact Reduction)

It is known that when reconstruction is performed on highly absorbing X-ray materials, such as metal, it is affected by a phenomenon called beam hardening correction, which creates artifacts around the material and affects the reading of the image [21]. The EXAVISTA system’s High-quality Metal Artifact Reduction (HiMAR) process extracts the metal part from the projection data and uses the extracted metal information to create metal-attenuated projection data. The HiMAR process reduces metal artifacts and allows for the observation of the bone and bone beams around the metal [20].

#### 2.2.3. Iterative Reconstruction

Iterative reconstruction is a method of creating tomographic images with less noise by comparing the noise components of the collected projection image and the projection image being updated, thereby correcting the errors successively while implementing noise reduction processing to separate noise and structures based on a statistical model. For example, by treating fractures, trabeculae, and highly absorbent materials, such as metallic structures, it is possible to obtain images with reduced noise. This process provides a clearer cross-sectional image without losing the delineation of fine fractures and trabeculae. Moreover, when applied together with HiMAR processing, it is possible to obtain a clearer image of the bone and trabeculae around the metal [20].

### 2.3. Implant

In this study, we used a fully HA-coated stem, the POLARSTEM, which is a titanium alloy (Ti-6AL-4V)-based material with a plasma spray coating of 180 μm pure titanium and 50 μm HA coating on the top surface. The compaction rasp compresses the trabecular bone during rasping, thereby preserving the trabecular bone around the stem. The triple tapered design of this stem is expected to provide rotational stability at the proximal femur, stem subsidence, and good initial fixation with proximal load transfer. OXINIUM (Smith & Nephew, Inc.) was used as the head. For the acetabular side, either the R3 Cup (Smith & Nephew, Inc.) or the SQRUM TT Cup (Kyocera, Kyoto, Japan) were used for both three-dimensional (3D) porous structures. The R3 cup was initially used; however, when the acetabular diameter was <46 mm, the SQRUM TT cup was selected from the multihole variations.

### 2.4. Surgical Procedure

In the 1 y group, 19 joints underwent the posterolateral approach and 1 joint underwent the direct lateral approach; in the 3 y group, 13 joints underwent the posterolateral approach and 7 joints underwent the direct lateral approach. We used 3D preoperative planning using the ZedHip (LEXI Co., Ltd., Tokyo, Japan) as a reference. The thickness of the preserved cancellous bone was adjusted at the discretion of the surgeon according to the fragility of the patient’s cancellous bone. A collarless stem was used in all joints in both groups.

### 2.5. Outcomes

Plain radiography and DTS at the time of the final observation were performed for imaging evaluation. Plain radiography was performed in two directions, the anterior–posterior (AP) view and the lateral view, and DTS was conducted in two directions, the AP view and Lauenstein projection using the EXAVISTA system. In both views, we attempted to obtain cross-sectional images parallel to the stem axis. In the case of the Lauenstein projection, the entire stem was imaged as a mild medial dislocation in a single tomogram. Plain radiography was used to evaluate RLs, cancellous condensation, cortical hypertrophy, and stress shielding. DTS was used to evaluate RLs, the presence of periprosthetic bone formation, and the thickness of the layer of bone formation. The thickness of bone formation was defined as the distance from the stem surface to the outside of the bone formation. The RL was defined as a linear shadow with a radiolucent image of at least 2 mm. Imaging evaluation was performed by a single hip surgeon.

### 2.6. Statistical Analysis

Statistical analysis was performed using SPSS Statistics (Version 28.0; IBM Corp., Armonk, NY, USA). Sex and primary disease parameters of the 1 y and 3 y groups were analyzed using Pearson’s chi-squared test. Comparison of age, body mass index (BMI), and the thickness of the bone formation between the 1 y and 3 y groups was performed using the Mann–Whitney U-test. In all analyses, a *p*-value of <0.05 was considered statistically significant.

## 3. Results

### 3.1. Patient Demographics

There were twenty joints in the 1 y postoperative group (five male and fifteen female patients, respectively; all patients had a postoperative observation period of 12 mo) and twenty joints in the 3 y postoperative group (one male and nineteen female patients, respectively; the mean postoperative observation period was 41.7 (range: 36–49) mo). The 1 y group had a mean age at surgery of 68.3 ± 9.7 (range: 52–84) y and a mean BMI of 22.9 ± 2.7 (range: 18–28). The 3 y group had a mean age at surgery of 68.8 ± 9.3 (range: 54–81) y and a mean BMI of 24.3 ± 3.8 (range: 16–30) (Table 1).

In the 1 y group, there were eighteen joints with developmental dysplasia of the hip and two joints with primary osteoarthritis; in the 3 y group, there were eighteen joints with developmental dysplasia of the hip, one joint with primary osteoarthritis, and one joint with rapidly destructive coxopathy. The Crowe classification [22] was Group 1 in all 20 joints in the 1 y group. Group 1 included sixteen joints, whereas Group 2 included four joints in the 3 y group. The distribution of the stem size used is shown in Table 2.

### 3.2. Plain Radiography

The Dorr classification was Types A, B, and C in 1, 17, and 2 cases for the 1 y group and Types A, B, and C in 3, 13, and 4 cases for the 3 y group, respectively. The CFI was 3.39 ± 0.54 and 3.79 ± 0.69 in the 1 y group and 3 y group, respectively.

No cases of an RL with osteopetrosis of 2 mm or more in either group were noted. Furthermore, cortical hypertrophy was not observed in either group. First- and second-degree stress shielding was observed in sixteen and four cases in the 1 y group and two and eighteen cases in the 3 y group, respectively. No cases of third-degree or higher stress shielding in either group were noted. Cancellous condensation was observed in two (10%) and fourteen (70%) patients in the 1 y group and 3 y group, respectively.

### 3.3. Digital Tomosynthesis

**Figure 4:**
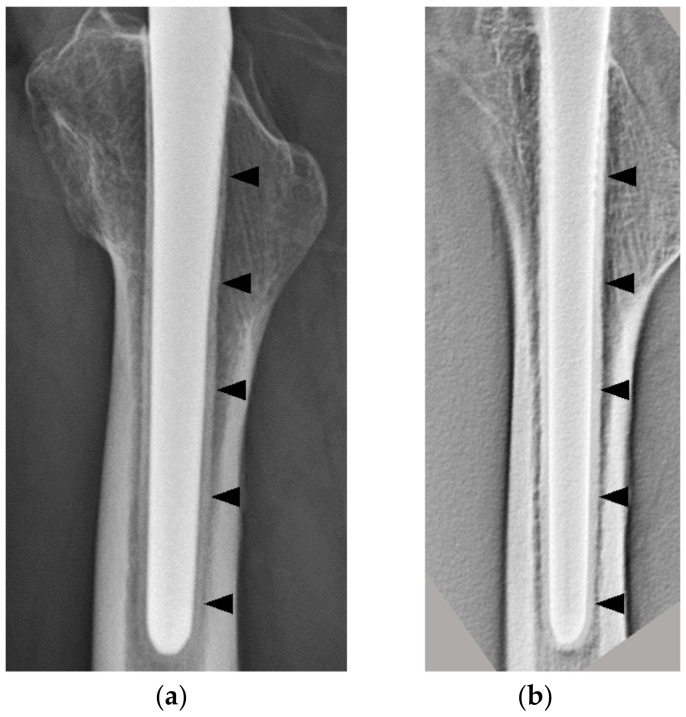
An 81-year-old female patient 3 y after THA. (**a**) Plain radiographs (lateral view); there was bone formation surrounding the stem (black arrowheads). (**b**) DTS (lateral view); in the same patient as in (**a**), bone formation was observed uniformly around the stem from proximal to distal (black arrowheads), and the stem–bone interface is seen more clearly in (**b**) than in (**a**). THA, total hip arthroplasty; DTS, digital tomosynthesis.

In all cases, bone formation was observed uniformly around the stem from proximal to distal (Figure 4).

**Figure 5:**
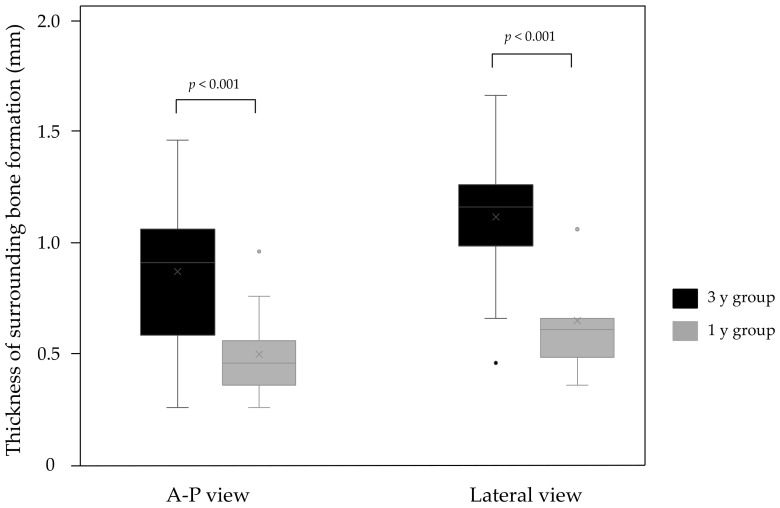
In both AP and lateral views, the 3 y group had a significantly thicker bone formation than the 1 y group (*p* < 0.001). AP, anterior–posterior.

In the AP view, the thicknesses of the osteogenic layer were 0.54 ± 0.19 and 0.91 ± 0.31 mm in the 1 y group and 3 y group (*p* < 0.001), respectively, and there was a significant difference. In the lateral view, the thicknesses of the osteogenic layer were 0.69 ± 0.20 and 1.16 ± 0.27 mm in the 1 y group and 3 y group (*p* < 0.001), respectively, and this was also significantly different (Figure 5). No RLs with osteopetrosis in either group were noted.

## 4. Discussion

Unlike the single tomogram obtained by general imaging with film, DTS acquires an arbitrary tomographic image in a single scan, reduces metal artifacts, and reconstructs the tomographic image using various algorithms [3,23,24]. In this study, we confirmed the osteogenic response around the stem using DTS. This is a layer of bone formation that occurs uniformly from proximal to distal on the surface of the HA-coated stem, and this reaction was defined as the surrounding bone formation (SBF).

In some cases, the SBF could not be seen on plain radiography, and in other cases, cancellous condensation was seen around the stem as a permeable line, which at first glance appeared as an RL (Figure 2). By contrast, on DTS, no radiolucent image was observed at the stem–bone interface in all cases, although the thickness of the SBF varied. Geesink et al., reported good results for HA-coated implants in biological fixation. Regarding the thickness of the HA coating, it has been reported that a thickness of 150 μm or more increases the risk of the coating peeling off due to fatigue fracture, while a thickness of 30–90 μm reduces the risk of fatigue fracture and allows the HA to function well. A thickness of 30–90 μm has been reported as a good thickness for the HA to work successfully while reducing the risk of fatigue failure [25]. Based on the above, the POLARSTEM adopted a 50 μm HA coating. Osseointegration is a process in which the lamellar bone adheres to the implant without fibrous tissue [26]. The micromotion of the stem is described as a fibrous tissue formation above 150 μm, mixed bone and fibrous tissue between 40 and 150 μm, and predominantly bone formation below 20 μm. Histological analysis has shown that the HA coating stabilizes the implant at an early stage and suppresses stem micromotion by promoting bone formation [26]. The results of the SBF observed in all patients in this study suggested that this is a stable stem response that does not result in micromotion. This may have a favorable impact on the long-term outcomes of the stem.

In some cases of fully HA-coated stems, cancellous condensation that occurs circumferentially on plain radiography may appear to be an RL at first glance and may be evaluated as aseptic loosening. The results of this study confirmed the usefulness of the detailed evaluation by DTS, especially in cases where an RL is suspected in fully HA-coated stems.

The POLARSTEM is a fully HA-coated stem and has a characteristic rasp shape that compresses the cancellous bone circumferentially to create a cancellous bone bed. Further, the triple-tapered stem design provides a strong initial fixation and is independent of the medullary cavity morphology and bone quality. The surface finish is a 180 μm titanium plasma coating with a 50 μm HA coating around the entire circumference, which is expected to enhance the osteoconductive effect of the HA for osseointegration and provide good long-term results. The long-term revision rate of the POLARSTEM is lower than that of other cementless stems, and the results have been reported to be good [5]. Good midterm results at 7 y postoperatively and long-term results at 11 y postoperatively have also been reported [27,28,29]. The SBF observed using DTS in the present study may reflect the effect of the HA coating and may contribute to the good long-term results of the POLARSTEM.

The patient cost for DTS is approximately twice compared to that of plain radiography, although less than one-third of that for CT [30,31,32]. Higher interobserver agreement has been reported with DTS than with radiography [33,34]. In this study, as shown in Figure 3, some cases of the SBF could be easily observed on plain radiographs. However, there were several cases that appeared to be stem loosening, and overestimation on plain radiographs has a significant impact on surgical outcomes and subsequent treatment strategies. Certainly, this does not change the usefulness of plain radiograph diagnosis, and the initial cost of installing a machine must be taken into consideration. However, we were able to demonstrate the usefulness of DTS as a diagnostic aid.

## 5. Limitation

This study has some limitations. First, it was a cross-sectional study, and the two groups compared were not the same patients. There is a need to compare the same patients over time in future studies. Second, imaging evaluations were performed by a single hip surgeon; therefore, no comparisons were made among multiple examiners. Third, it was a short-term study of only 3 y, and the effects of confirmed bone formation on the long-term prognosis remain unknown. Therefore, long-term follow-up, including regular DTS imaging, is necessary.

## 6. Conclusions

Our analysis suggested that DTS was able to diagnose bone formation around the stem used for total hip arthroplasty, which was difficult to confirm on plain radiography.

## Figures and Tables

**Table 1:** Baseline data ^1^ of the two groups.

	1 Y Group (*n* = 20)	3 Y Group (*n* = 20)	*p*-Value
Sex; male/female (*n*)	5/15	2/18	0.182
Mean BMI ^2^ (kg/m^2^)	22.9 ± 2.7	24.3 ± 3.8	0.188
Mean age (y)	68.3 ± 9.7	68.8 ± 9.3	0.874

^1^ Patients’ sex, BMI, and age were not significantly different between the 1 y and 3 y groups according to the chi-squared test and Mann–Whitney U-test. ^2^ BMI, body mass index.

**Table 2:** Stem size distribution.

Stem Size	1 Y Group (*n*)	3 Y Group (*n*)
0	5	2
1	3	6
2	5	4
3	3	5
4	2	2
5	1	0
6	0	1
7	1	0

## Data Availability

The datasets used and/or analyzed during the current study are available from the corresponding author upon reasonable request.

## References

[1] Blum A., Noël A., Regent D., Villani N., Gillet R., Teixeira P.G. (2018). Tomosynthesis in musculoskeletal pathology. Diagn. Interv. Imaging.

[2] Geijer M., Gunnlaugsson E., Götestrand S., Weber L., Geijer H. (2017). Tomosynthesis of the thoracic spine: Added value in diagnosing vertebral fractures in the elderly. Eur. Radiol..

[3] Machida H., Yuhara T., Mori T., Ueno E., Moribe Y., Sabol J. (2010). Optimizing Parameters for Flat-Panel Detector Digital Tomosynthesis. Radiographics.

[4] Göthlin J.H., Geijer M. (2013). The Utility of Digital Linear Tomosynthesis Imaging of Total Hip Joint Arthroplasty with Suspicion of Loosening: A Prospective Study in 40 Patients. BioMed Res. Int..

[5] Ben-Shlomo Y., Blom A., Boulton C., Brittain R., Clark E., Craig R., Dawson-Bowling S., Deere K., Esler C., Espinoza O. (2020). National Joint Registry Annual Reports. The National Joint Registry 17th Annual Report 2020.

[6] Bakkai A., Ryan P., Goga I. (2017). Tapered uncemented HA-coated femoral stems: A radiological study. SA Orthop. J..

[7] Vidalain J.-P. (2010). Twenty-year results of the cementless Corail stem. Int. Orthop..

[8] Duffy P., A Masri B., Garbuz D., Duncan C.P. (2006). Evaluation of patients with pain following total hip replacement. Instr. Course Lect..

[9] Patel A.R., Sweeney P., Ochenjele G., Wixson R., Stulberg S.D., Puri L.M. (2015). Radiographically Silent Loosening of the Acetabular Component in Hip Arthroplasty. Am. J. Orthop..

[10] Engh C.A., Massin P., Suthers K.E. (1990). Roentgenographic assessment of the biologic fixation of porous-surfaced femoral components. Clin. Orthop. Relat. Res..

[11] Miller T.T. (2012). Imaging of hip arthroplasty. Eur. J. Radiol..

[12] A Walde T., E Weiland D., Leung S.B., Kitamura N., Sychterz C.J., Engh A., Claus A.M., Potter H.G., A Engh C. (2005). Comparison of CT, MRI, and Radiographs in Assessing Pelvic Osteolysis. Clin. Orthop. Relat. Res..

[13] Zotti M.G., Campbell D., Woodman R. (2011). Detection of Periprosthetic Osteolysis Around Total Knee Arthroplasties. J. Arthroplast..

[14] Arachchi S., Pitto R.P., Anderson I.A., Shim V.B. (2015). Analyzing bone remodeling patterns after total hip arthroplasty using quantitative computed tomography and patient-specific 3D computational models. Quant. Imaging Med. Surg..

[15] Gillet R., Teixeira P., Bonarelli C., Coudane H., Sirveaux F., Louis M., Blum A. (2018). Comparison of radiographs, tomosynthesis and CT with metal artifact reduction for the detection of hip prosthetic loosening. Eur. Radiol..

[16] Guo S., Tang H., Zhou Y., Huang Y., Shao H., Yang D. (2018). Accuracy of Digital Tomosynthesis With Metal Artifact Reduction for Detecting Osteointegration in Cementless Hip Arthroplasty. J. Arthroplast..

[17] Oishi K., Yamamoto Y., Harada Y., Inoue R., Sasaki E., Ishibashi Y. (2021). Radiographic assessment of radiolucent lines around a highly porous titanium cup (Tritanium) using digital tomosynthesis, after total hip arthroplasty. J. Orthop. Surg. Res..

[18] Tang H., Huang X., Cheng X., Yang D., Huang Y., Zhou Y. (2020). Evaluation of peri-prosthetic radiolucent lines surrounding the cementless femoral stem using digital tomosynthesis with metal artifact reduction: A cadaveric study in comparison with radiography and computed tomography. Quant. Imaging Med. Surg..

[19] Noble P.C., Alexander J.W., Lindahl L.J., Yew D.T., Granberry W.M., Tullos H.S. (1988). The anatomic basis of femoral component design. Clin. Orthop. Relat. Res..

[20] Otake S. (2018). Tomosynthesis Imaging Application of the EXAVISTA General-purpose Radiography and Fluoroscopy Table System—Usefulness in the Orthopedic Field. MEDIX.

[21] Kyriakou Y., Meyer E., Prell D., Kachelriess M. (2010). Empirical beam hardening correction (EBHC) for CT. Med. Phys..

[22] Crowe J.F., Mani V.J., Ranawat C.S. (1979). Total hip replacement in congenital dislocation and dysplasia of the hip. J. Bone Jt. Surg.-Am. Vol..

[23] Canella C., Philippe P., Pansini V., Salleron J., Flipo R.-M., Cotten A. (2011). Use of Tomosynthesis for Erosion Evaluation in Rheumatoid Arthritic Hands and Wrists. Radiology.

[24] Hayashi D., Xu L., Roemer F.W., Hunter D.J., Li L., Katur A.M., Guermazi A. (2012). Detection of Osteophytes and Subchondral Cysts in the Knee with Use of Tomosynthesis. Radiology.

[25] Geesink R.G., De Groot K., Klein C.P. (1987). Chemical implant fixation using hydroxyl-apatite coatings. The development of a human total hip prosthesis for chemical fixation to bone using hydroxyl-apatite coatings on titanium substrates. Clin. Orthop. Relat. Res..

[26] Albrektsson T., Brånemark P.-I., Hansson H.-A., Lindström J. (1981). Osseointegrated Titanium Implants:Requirements for Ensuring a Long-Lasting, Direct Bone-to-Implant Anchorage in Man. Acta Orthop. Scand..

[27] Lee P.Y., Evans A.R. (2014). Early Failure of the Polarstem Total Hip Arthroplasty—Can The Australian NJR Tell Us The Full Story?. J. Arthroplast..

[28] Assaf A., Manara J.R., Teoh K.H., Evans A.R. (2019). Mid-term clinical results of the cementless R3 cup and Polarstem total hip arthroplasty. Eur. J. Orthop. Surg. Traumatol..

[29] Willburger R.E., Heukamp M., Lindenlaub P., Efe T., Peterlein C.-D., Schüttler K.-F. (2020). Excellent midterm survival and functional outcomes of a fully hydroxyapatite-coated cementless stem: First results of a prospective multicenter study. Arthroplast. Today.

[30] Båth M., Svalkvist A., von Wrangel A., Rismyhr-Olsson H., Cederblad Å. (2010). Effective dose to patients from chest examinations with tomosynthesis. Radiat. Prot. Dosim..

[31] Koyama S., Aoyama T., Oda N., Yamauchi-Kawaura C. (2010). Radiation dose evaluation in tomosynthesis and C-arm cone-beam CT examinations with an anthropomorphic phantom. Med. Phys..

[32] Huppertz A., Radmer S., Asbach P., Juran R., Schwenke C., Diederichs G., Hamm B., Sparmann M. (2011). Computed tomography for preoperative planning in minimal-invasive total hip arthroplasty: Radiation exposure and cost analysis. Eur. J. Radiol..

[33] Ottenin M.-A., Jacquot A., Grospretre O., Noel A., Lecocq S., Louis M., Blum A. (2012). Evaluation of the Diagnostic Performance of Tomosynthesis in Fractures of the Wrist. Am. J. Roentgenol..

[34] Von Steyern K.V., Björkman-Burtscher I., Höglund P., Bozovic G., Wiklund M., Geijer M. (2012). Description and validation of a scoring system for tomosynthesis in pulmonary cystic fibrosis. Eur. Radiol..

